# A multiplexed microfluidic continuous-flow electroporation system for efficient cell transfection

**DOI:** 10.21203/rs.3.rs-3538613/v1

**Published:** 2023-11-07

**Authors:** Jacob A. VanderBurgh, Grant T. Corso, Stephen L. Levy, Harold G. Craighead

**Affiliations:** CyteQuest, Inc; CyteQuest, Inc; CyteQuest, Inc; CyteQuest, Inc

**Keywords:** Transfection, cell therapy, immunotherapy, non-viral, electroporation, cancer

## Abstract

Cellular therapies have the potential to advance treatment for a broad array of diseases but rely on viruses for genetic reprogramming. The time and cost required to produce viruses has created a bottleneck that constricts development of and access to cellular therapies. Electroporation is a non-viral approach for genetic reprogramming that bypasses these bottlenecks, but current electroporation technology suffers from low throughput, tedious optimization, and difficulty scaling to large-scale cell manufacturing. Here, we present an adaptable microfluidic electroporation platform with the capability for rapid, multiplexed optimization with 96-well plates. Once parameters are optimized using small volumes of cells, transfection can be seamlessly scaled to high-volume cell manufacturing without re-optimization. We demonstrate optimizing transfection of plasmid DNA to Jurkat cells, screening hundreds of different electrical waveforms of varying shapes at a speed of ~3 s per waveform using ~ 20 μL of cells per waveform. We selected an optimal set of transfection parameters using a low-volume flow cell. These parameters were then used in a separate high-volume flow cell where we obtained similar transfection performance by design. This demonstrates an economical method for scaling to the volume required for producing a cell therapy without sacrificing performance.

## 1. Introduction

Cellular therapies have the potential to advance treatment for a broad array of diseases and are composed of living cells that have been genetically engineered as the drug administered to patients. For instance, chimeric antigen receptor (CAR)-T cell therapies have shown remarkable efficiency in certain hematological cancers [1]–[5]. Currently approved CAR-T cell therapies consist of autologous T cells genetically reprogrammed to express a CAR, which fuses an extracellular antigen-binding domain with intracellular signaling modules [6]. By selecting the target antigen, CAR-T cells can be engineered to specifically target and destroy cancer cells. Since the first FDA approved treatment in 2017 for acute lymphoblastic leukemia, the number of studies focused on CAR-T cell therapies has grown exponentially with an estimated 2,129 CAR-T cell therapies being developed as of January 2023 [7]. Currently approved CAR-T cell therapies utilize lentivirus or adeno-associated virus (AAV) for genetic engineering, but viruses have several drawbacks related to their relatively lengthy manufacturing processes, immunogenicity, and potential for insertional mutagenesis [8]–[10]. In particular, the time and expense required to manufacture viral products, coupled with the explosive growth in the cell therapy field, has created a bottleneck in which demand far outstrips supply.

The viral bottleneck has led to intense efforts to develop non-viral methods for genetic engineering [11]. Electroporation is a well-known method for delivery of DNA, RNA, and proteins into cells, that is recognized as a leading replacement for viral delivery [11]–[13]. During electroporation, a time-varying electric field creates transient pores in the cell membrane to allow molecules to diffuse into cells [14]–[16]. Electroporation can engineer target cells with specialized cargo such as plasmid DNA or CRISPR-Cas9 ribonucleoproteins (RNPs) to manufacture cells for cell therapies. These cargos are simpler and cheaper to manufacture relative to viruses, and they can be adapted more quickly during research and development to screen potential drug candidates. Thus, electroporation has the potential to bypass viral bottlenecks in cell therapy.

Despite the known potential, electroporation has not been widely adopted because current equipment has technical limitations that make essential process development difficult. Most commercial electroporation systems are “batch-at-a-time” systems in which a fixed “batch” of cells and cargo are electroporated in a container, such as a cuvette, with an electric pulse. For example, cells mixed with a given concentration of plasmid DNA could be electroporated with a simple square wave pulse defined by a pulse voltage, pulse duration, and number of pulses. These parameters (voltage, duration, pulse number, and plasmid DNA concentration) are empirically varied to balance transfection efficiency against cell viability [17], [18]. However, this optimization can be time-consuming because of the large number of parameters to vary. Also, transferring cell solutions between a large number of cuvettes is labor intensive. These systems also offer limited flexibility in the cell processing volume which must be matched to the cuvette size. Importantly, as the cuvette size is altered to accommodate larger volumes of cells, the gap between the electrodes and the electric field experienced by the cell is also altered. As such, cuvette-based systems are unsuitable to scale for the high number of cells required for therapies, such as a typical CAR-T cell therapy which requires greater than 1 billion cells per dose [11], [19], [20]. The comparatively few commercial electroporation devices available for large-volume cell manufacturing also rely on cuvettes for low-volume optimization, limiting their ability to efficiently screen transfection parameters and scale these parameters for high-volume manufacturing. Overall, there is a need for a flexible electroporation platform that can easily optimize transfection parameters using low volumes of cells and reagents, and once identified, apply these parameters for large-volume cell manufacturing without re-optimization.

Here, we describe a microfluidic electroporation platform that provides flexible, multiplexed, and efficient optimization capabilities with ease of scaling from low-volume optimization to high-volume cell manufacturing. Flexibility and scaling capabilities are afforded by the microfluidic electroporation chip, which we have previously shown can be scaled for any desired range of cell volume without changing the electric field experienced by the cells [21]. Here, we take advantage of this capability by optimizing transfection efficiency using small volumes of cells using flow chips with 2 mm channel widths. We demonstrate multiplexed optimization by simultaneously screening 8 different plasmid concentrations and 9 different electrical waveforms in a 96-well plate. After selecting one plasmid concentration, we rapidly screened hundreds of different voltage waveforms of varying shapes, durations, and amplitudes using ~ 3 s and ~ 20 μL of cells per waveform. Finally, we selected one set of optimized transfection parameters and scaled our processing throughput from 1.6 million cells/minute to 8 million cells/minute by scaling our flow chip’s channel width from 2 mm to 10 mm without impacting the electric field experienced by the cells, producing similar transfection performance at both rates. We believe these data demonstrate how our platform could enable electroporation to be more broadly adopted by researchers and manufacturers seeking a non-viral method to engineer cells for cellular therapies.

## 2. Electroporation platform overview

**Figure 1:**
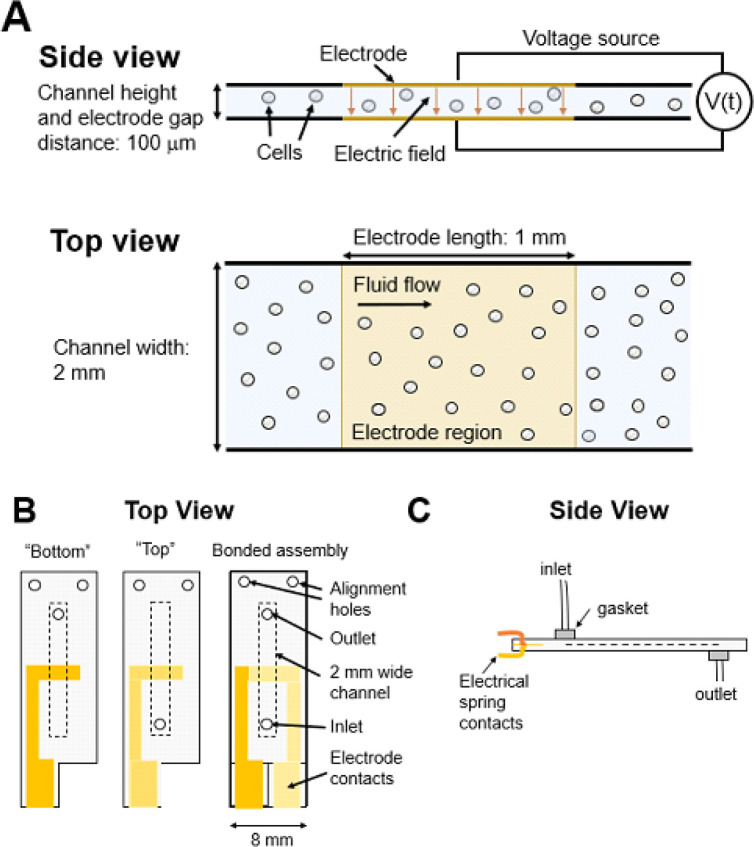
Schematic of flow chip. (A) Top and side view schematic of microfluidic channel. Cells experience a spatially uniform and temporally varying electric field region between parallel gold electrodes. By fixing the channel height and changing the channel width, the flow chip can be designed to match the desired volumetric throughput without changing the electric field or chemical environment experienced by the cells. Approximately to-scale schematic. (B) Top and (C) side view schematic of the entire flow chip.

Our platform incorporates a microfluidic flow chip that has a thin slab geometry which is the basis for our platform’s reproducibility, high degree of flexibility, and easy scaling between processing speeds. The flow chip’s channel height, and the distance between the parallel gold electrodes is 100 μm (Fig. 1A). The parallel-plate geometry results in a spatially uniform electric field in the region between the electrodes and ensures that each cell is subject to the same electric field enabling reproducible electroporation. The thin gap between the electrodes also permits us to generate electric field strengths necessary for producing transient pore formation in the plasma membrane, typically estimated between 10–100 kV/m [22], using relatively low voltage amplitudes (1–10 V). In contrast, the large gaps between electrodes in cuvettes (e.g. 2–4 mm) typically seen in commercial systems require a much higher voltage, which limits their flexibility for generating voltage waveforms. Operating with low voltages provides us with the flexibility to generate arbitrary time-varying voltage waveforms, not just simple high-voltage DC pulses.

The flow chip’s channel width is much larger than its height to allow for rapid processing speeds. Critically, the width of the flow chip can be selected to match the desired processing speed without changing the electric field experienced by the cells. This principle is due to the thin slab geometry of the flow chip, in which the ratio of the thin (height) to transverse (width) dimension is less than 10%. This results in the fluid velocity profile in a rectangular chip becoming ‘plug-shaped’ along the wide dimension, minimizing the variation in the time each cell is subject to the electric field [23]. As such, optimization can be performed in a flow chip with a small channel width (e.g. 2 mm) to determine transfection parameters using small volumes of cells and reagents. After optimization, we can use a different flow chip with the same channel height but a larger channel width (e.g. 10 mm) to increase volumetric throughput without re-optimizing transfection parameters.

**Figure 2:**
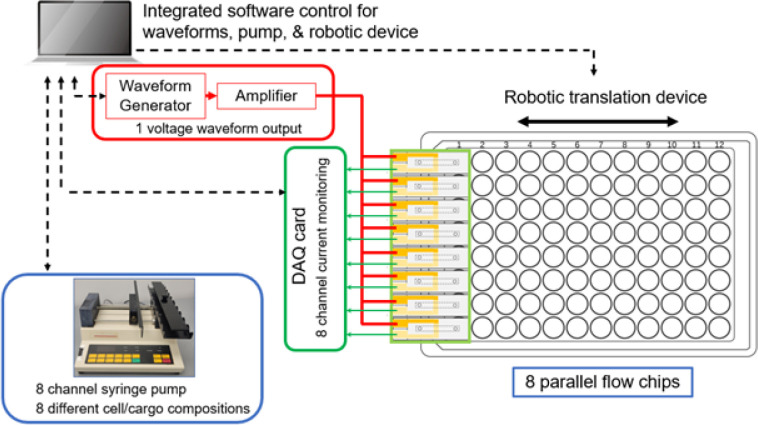
Schematic of major components comprising electroporation platform.

**Figure 3:**
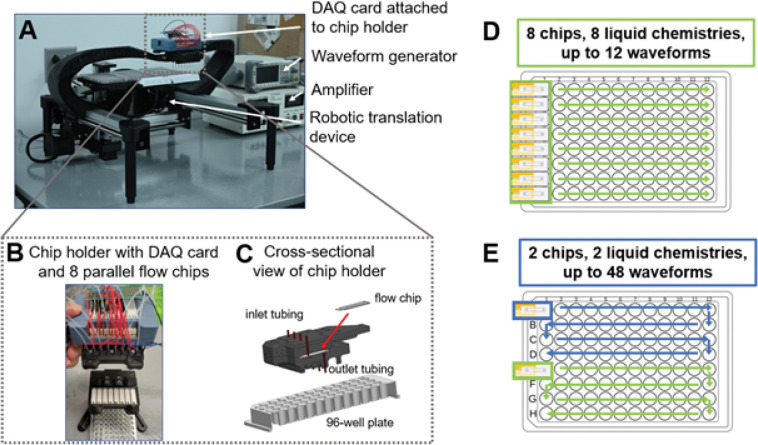
Photographs and schematics of electroporation platform and operation modes. (A) Photograph of electronics, chip holder, and robotic translation device. (B) Photograph of chip holder that includes 8 parallel flow chips, embedded gaskets, attached tubing, and DAQ card with electrical connections to both apply voltage waveforms and measure the voltage drop across 10 Ω resistors in series with each flow chip. (C) Cross-sectional schematic view of chip holder that depicts how inlet and outlet tubing attach to flow chip. (D) Experimental setup for simultaneous 8-channel or (E) 2-channel optimization with arrows indicating the travel path of the robotic translation device.

The flow chip is designed as a single-use consumable that has fluid inlet/outlets to receive cells suspended in electroporation buffer containing the cargo to be delivered (Fig. 1B–C). Our system consists of up to 8 flow chips with an 8-channel syringe pump, electronics to generate and amplify voltage waveforms, a data acquisition (DAQ) card to measure voltages, and automated liquid handling (Fig. 2). The flow chips fit in a custom-built chip holder, with spacing designed to match the pitch of a 96-well plate for high-throughput screening (Fig. 3A–C). During operation, cells suspensions are mixed with their cargo, loaded into 1–8 syringes, and pumped into each channel by the syringe pump. We previously programmed and uploaded time varying voltage waveforms into a function generator. A waveform is applied continuously so that cells flowing through the chip experience a time-varying voltage when they are situated between the electrodes. We use a DAQ card to measure the current by measuring the voltage dropped across a 10-ohm resistor in series with the flow chip. The resistance of the flow chip is approximately 5.68 kΩ. As cells exit the channel through outlet tubing, our robotic translation device dispenses cells into a multi-well plate, with the option of loading up to six multi-well plates. The activities of the syringe pump, function generator, DAQ card, and robotic translation device are controlled by a python script.

Before an experiment, the user selects the number of parallel channels/liquid compositions and the number of waveforms to test. The fluid flow is continuous during the run while separate voltage waveforms may be applied in coordination with the movement of the well plate. For example, a user could choose to screen 8 different liquid chemistries (e.g. 8 different cargo concentrations). In this case, when using a single 96-well plate, each of the 8 rows of the 96-well plate receives cells from each liquid composition, while each of the 12 columns of the 96-well plate receives cells treated with different voltage waveforms (Fig. 3D). Alternatively, a user could choose to screen 2 different liquid chemistries and screen 48 different voltage waveforms (Fig. 3E). By minimizing the volume downstream of the electrodes, we can ensure that when the voltage waveform is altered, cells exiting the outlet tube will rapidly represent cells that have been subject to the latest waveform. As a result, our platform enables rapid and multiplexed scanning of transfection parameters using small volumes of cells and reagents to select optimal electroporation conditions.

## 3. Materials and methods

### 3.1 Fabrication of electroporation flow chips

Electroporation flow chips were constructed from a three-layer stack of polymer substrates as described previously [21]. Briefly, gold with a chromium adhesion layer was vapor deposited onto either acrylic or PET plastic and patterned to create gold electrodes on the top and bottom layers of the flow chips. The middle layer was composed of a thin, laser-cut, hydrophilic, pressure sensitive, adhesive tape. The removed area of tape defined the channel, while the thickness defined the channel height. Flow chips were fabricated by bonding the three-piece sandwich assembly through compression in a press.

### 3.2 Cell culture, reagents, and DNA constructs

Jurkat cells were purchased from Millipore Sigma (Millipore Sigma, Burlington, MA, USA) and cultured in RPMI 1640 medium supplemented with 10% fetal bovine serum (R&D Systems, Minneapolis, MN, USA). All cells were maintained at 37 °C and 5% CO_2_. pEF1a-GFP (plasmid DNA; 2473 bp) was purchased from Aldevron (Fargo, ND, USA).

### 3.3 Electroporation procedure

Cells were harvested from flasks, counted, and washed two times in BTXpress Cytoporation low-conductivity electroporation buffer (Conductivity: 9 × 10^−3^ S/m; Holliston, MA, USA). After washing, cells were resuspended at 5 × 10^5^ cells/mL, mixed with plasmid DNA, and loaded into syringes. For 8-channel experiments, we used a Harvard Apparatus 22 Infusion Pump, while for 1- or 2-channel experiments we used a Harvard Apparatus PHD Ultra Infuse/Withdraw Pump (Holliston, MA, USA). Cell suspensions were flowed continuously into the flow chips at the indicated flow rates. As cells pass through the electrodes, they received a continuously cycling, time-varying arbitrary waveform generated by a function generator (Siglent SDG 1032X; Siglent Technologies, Solon, OH, USA) and amplified by a RF amplifier (TS250; Accel Instruments, Irvine, CA, SA). The voltage waveforms were monitored by an oscilloscope (Siglent SDS 1104X-E, Siglent Technologies). The current in up to 8-flow chips was monitored by a DAQ card by measuring the voltage dropped across a 10-ohm resistor in series with the flow chip (MCC USB 205, Digilent, Pullman, WA, USA). Cells exited the channel via outlet tubing, entering a well containing pre-warmed cell culture media. During an experiment, a custom-built robotic translation device moved the plate to dispense cells into a multi-well plate. The activities of the syringe pump, function generator, DAQ card, and robotic translation device were controlled by a python script.

### 3.4 Flow cytometry

Transfection performance was measured 24-h post-transfection by flow cytometry using a ZE5 Cell Analyzer (Bio-Rad, Hercules, CA, USA). Cells were prepared for flow analysis by first staining cells with the viability dye, 7-AAD, and incubating for 10 minutes (Fisher Scientific, Hampton, NH, USA). During analysis, cells were distinguished from cell debris through forward scatter (FSC) vs. side scatter (SSC) area plots. Single cells were then gated through FSC-area vs. FSC-height plots. Cell viability was measured by gating 7-AAD negative (live) and 7-AAD positive (dead) single cell populations. GFP expression was measured by gating viable cells that were GFP positive relative to zero-voltage controls. Representative flow cytometry plots and microscopic images from zero-voltage and electroporated cells are shown in Figures S1–2 of Online Resource 1.

### 3.5 Sample size and data analysis

Sample size is provided in the figure legends. All analyses and plots were completed with GraphPad Prism 10 (GraphPad Software Inc, La Jolla, CA, USA).

## 4. Results and discussion

### 4.1 Multiplexed optimization of cargo concentration

**Figure 4:**
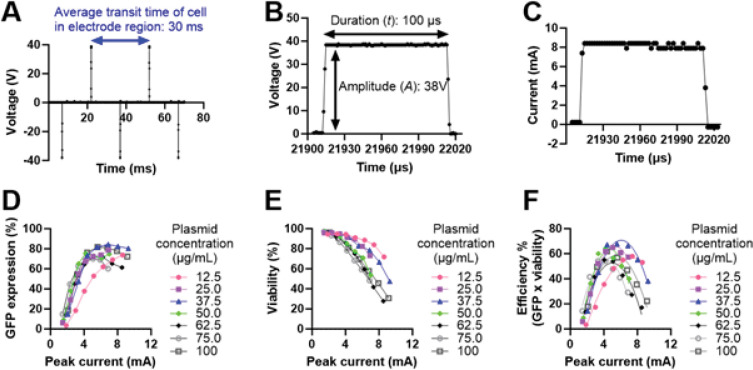
Multiplexed optimization of plasmid concentration. (A) Multiple cycles of the time-varying voltage applied to the electrodes. The waveform shown is a bipolar rectangular waveform with frequency (*f*) = 33 Hz, amplitude (*A*) = 38V, and duration (*t*) = 100 μs. (B) Zoomed time region of the applied V(t) waveform, depicting the voltage applied and (C) current through the channel. (D) Impact of varying peak current through the channel and plasmid concentration on GFP expression, (E) viability, and (F) efficiency (GFP expression x viability). Data in (F) are individual values fit to a second-order polynomial curve-fit.

To demonstrate the capability of our platform to perform multiplexed optimization, we transfected Jurkat cells with differing concentrations of plasmid DNA encoding GFP. Jurkat cells were resuspended at a concentration of 5 × 10^6^ cells/mL in low conductivity buffer containing plasmid DNA at concentrations ranging from 0 to 100 μg/mL, loaded into 8 syringes, and flowed into 8 electroporation flow chips at 400 μL/min. The waveform frequency, *f*, was set at 33 Hz resulting in cells receiving one bipolar waveform on average during their transit time as calculated by the cells’ average linear velocity, inferred from the volumetric flow rate and chip dimensions. Based on our previously published data, we initially screened bipolar rectangular waveforms with durations (*t*) of 100 μs and voltage amplitudes (*V*) between 10 V and 40 V [21]. Representative plots of the time-varying applied voltage and current through the channel are shown in Fig. 4A–C. We calculated the “peak current” through the channel as the average current value while the electrode was energized. We observed variation in the voltage-current relationship measured in each of the 8 different plasmid concentration conditions. For instance, for an applied voltage of 10 V, the peak current was 1.76 ± 0.28 mA. This standard deviation value indicates slight variations in the channel resistance, which can likely be attributed to small differences in the channel height and solution conductivity.

To measure transfection performance, we measured GFP expression and viability 24-h post-transfection using flow cytometry as described in our methods. Briefly, viability was measured using a viability dye (7-AAD) and calculated as the number of viable cells divided by the total number of cells. GFP expression was then calculated as the number of viable and expressing cells divided by the number of viable cells. We found that GFP expression tended to increase with increasing peak current and plasmid concentration, but that expression plateaued at ~ 80%. Similar to our previous report, we observed diminishing returns in GFP expression with increasing plasmid concentration. GFP expression increased significantly from 12.5 μg/mL to 25 μg/mL, but higher plasmid concentrations produced much smaller gains in GFP expression [21]. Viability decreased with increasing peak current and plasmid concentration. Efficiency, calculated as a single metric to balance the inverse relationship between GFP expression and viability, exhibited a quadratic relationship with respect to peak current. This quadratic relationship occurs because GFP expression plateaus with increasing current while viability monotonically decreases with increasing current. For all plasmid concentrations tested, efficiency peaked at an intermediate current value, although that peak occurred at different values of current for each plasmid concentration. Notably, we identified that an intermediate plasmid concentration, 37.5 μg/mL, exhibited a peak efficiency value of 68% at an intermediate peak current value of 5.6 mA. These results demonstrate how our platform can be used to perform rapid, multiplexed optimization of up to 8 channels.

### 4.2 Large-scale optimization of waveform shape

**Figure 5:**
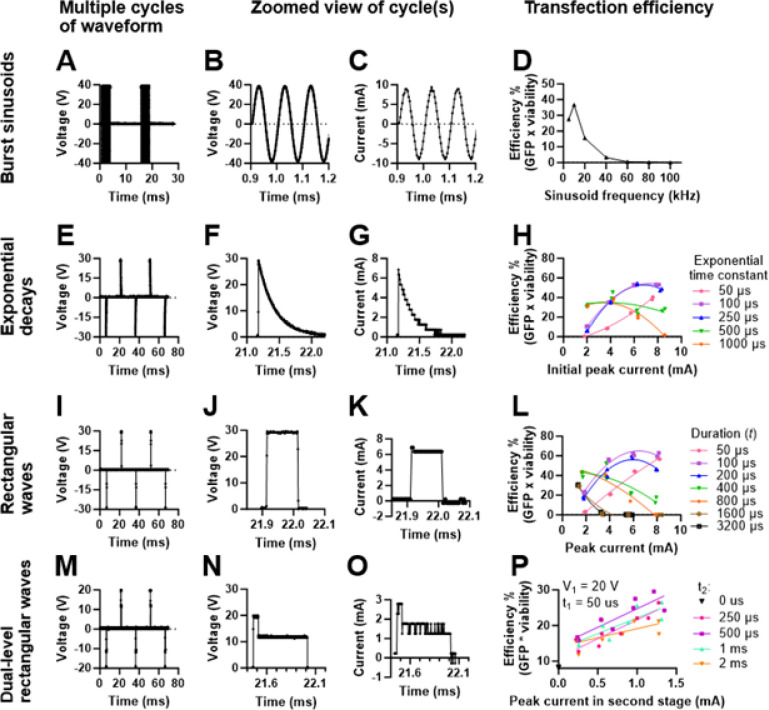
Optimization of voltage waveform shape. (A) Multiple cycles of a 3 ms burst sinusoid with a sine frequency of 10 kHz and 40 V amplitude. (B) Zoomed views of ~3 cycles of the voltage and (C) current channels. (D) Transfection efficiency plotted as a function of sinusoid frequency. (E) Multiple cycles of an exponential decay with a peak amplitude of 30 V and time constant of 250 μs. (F) Zoomed views of 1 positive cycle of the voltage and (G) current channels. (H) Transfection efficiency plotted as a function of initial peak current and exponential time constant. (I) Multiple cycles of a rectangular waveform with a duration of 100 μs and amplitude of 30 V. (J) Zoomed views of 1 positive cycle of the voltage and (K) current channels. (L) Transfection efficiency plotted as a function of average current and duration. (M) Multiple cycles of a dual-level rectangular waveform with a first level duration and amplitude of 50 μs and 20 V, and a second level duration and amplitude of 500 μs and 12 V. (P) Transfection efficiency plotted as a function of the second level’s average current and duration. Data in (D), (H), (L), and (P) collected during one optimization experiment in duplicate (2 values per voltage waveform). Data in (D) are mean ± SD, error bars are too small to be seen. Data in (H), and (L) are fit with second-order polynomial curve fits. Data in (P) are fit with a linear regression.

We tested the ability of our platform to generate an arbitrary waveform of any shape, and how this could improve transfection performance. We tested four bipolar waveform shapes, including sinusoidal bursts (Fig. 5A–C), exponential decays (Fig. 5E–G), rectangular waves (Fig. 5I–K), and dual-level rectangular waves (Fig. 5M–O). These waveform shapes were selected because they have previously been used by other groups during electroporation, but we note our platform is not limited to the waveforms shown here and could apply most arbitrary waveform shapes. [21], [24]–[28]. For our initial screening experiment, we tested 79 waveforms in duplicate, using two channels (two chips) with the robot travel pattern shown in Fig. 3E. Due to the number of waveform shapes under consideration, we screened a subset of the parameter space for each waveform shape. We selected these parameters, shown in Table I, based on data from previous experiments in our system (data not shown). Similar to our multiplexed experiment, we selected an overall waveform frequency of 33 Hz such that cells received 1 bipolar waveform on average during their transit under the electrodes.

Jurkat cells were prepared as described previously, and we selected a plasmid concentration of 37.5 μg/mL based on the results from our multiplexed optimization experiment. Cells were analyzed 24-h post-transfection using flow cytometry as described previously. Exponential decays (Fig. 5D) and rectangular waves (Fig. 5L) provided the highest values for efficiency (GFP expression x viability) with values of ~ 60%. However, we did not screen a large parameter-space for either sinusoid bursts (Fig. 5D) or dual-level rectangular waves (Fig. 5P). Overall, these results depict the flexibility of our electronics to deliver an arbitrary waveform.

We chose to further test dual-level rectangular waves because previous groups have found they can provide a favorable balance between GFP expression and viability [24], [25]. Since we observed minimal (< 5%) chip to chip variation while testing waveforms in duplicate, we swapped to testing waveforms with a single channel and tested up to 300 waveforms in a single experiment. At a rate of ~ 3 s per waveform, we could screen at a rate of ~ 20 waveforms per minute and we tested 572 waveforms in total (representative data from 80 waveforms is shown in a spreadsheet given in Online Resource 2). This throughput, which is performed with minimal user interaction, represents a significant advance over conventional cuvette-based electroporation systems. For instance, pipetting solutions between cuvettes is labor intensive, requiring ~ 20–30 s per cuvette, limiting the number of waveform parameters that can be realistically screened during optimization. Importantly, by using a small volume (~ 20 μL) of cell solution per waveform, we limit the consumption of expensive cells and reagents. Ultimately, the best performing waveform was a dual-level rectangular wave (V_1_ = 20V, t_1_ = 200 μs, V_2_ = 16V, t_2_ = 100 μs) that provided 84% GFP expression, 89% viability, and an efficiency of 74%. Overall, these results demonstrate how we can rapidly screen hundreds of waveforms to optimize transfection performance within a large parameter space.

### 4.3 Scale-up of processing throughput using optimized transfection parameters

**Figure 6:**
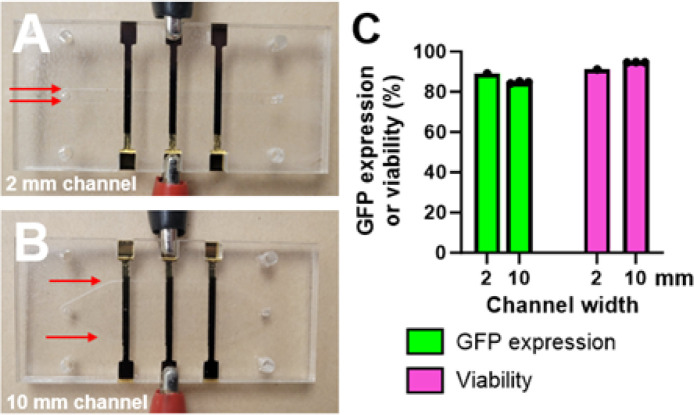
Scaling up processing throughput by increasing flow chip’s channel width. (A) Photographs of a flow chip with a 2 mm or (B) 10 mm channel width. Both channels have a channel height of 80 μm. Although there are three sets of independently addressable electrodes, the alligator clips indicate how one electrode pair is energized during an experiment. (C) GFP expression and viability data from Jurkat cells transfected with the same bipolar, dual-level rectangular waveform (V_1_ = 14V, t_1_ = 300 μs, V_2_ = 13V, t_2_ = 50 μs). Data from 2 mm channel width is one value while data from 10 mm channel is three duplicate wells collected during one experiment. Individual data points are plotted as circles overlaid on the mean value.

Finally, we tested the ability for our platform to scale processing throughput by proportionally increasing the channel width and volumetric flow rate. If the channel height is kept constant, proportionally increasing the volumetric flow rate and channel width leaves the cells’ average linear flow velocity unchanged, which results in cells experiencing the same electrical conditions. Using channels of 100 μm channel height, we tested scaling from channel widths of 2 mm to 10 mm and corresponding volumetric flow rates of 320 μL/min to 1.6 mL/min (Fig. 6A–B). For scale-up experiments, we swapped from the flow channels shown in Fig. 1B–C, which have a channel height of 80 μm, to those shown in Fig. 6A–B, which have a channel height of 100 μm, because we have not yet constructed 10 mm flow chips that have a channel height of 100 μm.

Jurkat cells were prepared as described previously using the same plasmid concentration of 37.5 μg/mL selected from the multiplexed optimization experiment. Cells were analyzed 24-h post-transfection using flow cytometry as described previously. Waveform frequency was similarly selected such that cells receive on average 1 bipolar waveform during their transit under the electrodes. We first performed a limited optimization with the new 2 mm channel, screening 216 bipolar dual-level rectangular waveforms, with corresponding data shown in a spreadsheet given in Online Resource 3. We selected the waveform with the highest efficiency (V_1_ = 14V, t_1_ = 300 μs, V_2_ = 13V, t_2_ = 50 μs), which produced 89% GFP expression, 91% viability, and 81% efficiency, and transfected cells in the 10 mm channel with the same waveform at 1.6 mL/min. Notably, we found that GFP expression and viability values were less than 5% different, with 85 ± 0.6% GFP expression, 95 ± 0.1% viability, and 80 ± 0.5% efficiency (Fig. 6C). Representative flow cytometry plots and microscopic images of control or electroporated Jurkat cells at this expression level are shown in Figures S1–2 in Online Resource 1. These results demonstrate how optimized transfection parameters can be directly translated for scaled-up processing speeds without re-optimization for similar performance.

## 5. Conclusions

Our continuous-flow, microfluidic electroporation platform provides advantages over current systems for optimizing transfection with flexibility in waveform design, up to 8 simultaneous channels, rapid screening, and low consumption of cells and reagents. Once transfection parameters have been optimized, it is simple to scale our chip’s geometry for producing high volumes of cells. These capabilities demonstrate an economical method to identify optimized transfection parameters and scale production to the volume required for cell therapies.

## Figures and Tables

**Table I:** Parameter-space screened during optimization of waveform shape.

Waveform shape	Parameters screened	Voltage and current plots
Sinusoidal bursts	Sine frequency: 5–100 kHz Burst duration: 3 ms Amplitude: 40 V	Figure 5A–C
Exponential decays	Time constant: 50 μs – 1 ms Initial peak amplitude: 10–40 V	Figure 5E–G
Rectangular waves	Duration: 50 μs – 3.2 ms Amplitude: 10–40 V	Figure 5I–K
Dual-level rectangular waves	Duration of 1st level: 50 μs Voltage of 1st level: 20–30 V Duration of 2nd level: 250 μs – 2 ms Voltage of 2nd level: 3–12 V	Figure 5M–O

## Data Availability

The data that support the findings of this study are available from the corresponding author, HGC, upon reasonable request.
